# New Therapeutics Options for Pediatric Neuromuscular Disorders

**DOI:** 10.3389/fped.2020.583877

**Published:** 2020-11-23

**Authors:** Marina Flotats-Bastardas, Andreas Hahn

**Affiliations:** ^1^Department of Pediatric Neurology, Saarland University Hospital, Homburg, Germany; ^2^Department of Child Neurology, University of Giessen, Giessen, Germany

**Keywords:** Duchenne and Becker muscular dystrophies, spinal muscular atrophies, Pompe disease, Zolgensma, Spinraza, AAV (adeno-associated virus)

## Abstract

Neuromuscular disorders (NMDs) of Childhood onset are a genetically heterogeneous group of diseases affecting the anterior horn cell, the peripheral nerve, the neuromuscular junction, or the muscle. For many decades, treatment of NMDs has been exclusively symptomatic. But this has changed fundamentally in recent years due to the development of new drugs attempting either to ameliorate secondary pathophysiologic consequences or to modify the underlying genetic defect itself. While the effects on the course of disease are still modest in some NMDs (e.g., Duchenne muscular dystrophy), new therapies have substantially prolonged life expectancy and improved motor function in others (e.g., spinal muscular atrophy and infantile onset Pompe disease). This review summarizes recently approved medicaments and provides an outlook for new therapies that are on the horizon in this field.

## Introduction

Neuromuscular disorders (NMDs) include conditions affecting the anterior horn cell (e.g., Spinal muscular atrophy = SMA), the peripheral nerve (e.g., Charcot-Marie-Tooth disease = CMT), the neuromuscular junction (e.g., Congenital myasthenia), or the muscle itself (e.g., Duchenne muscular dystrophy = DMD). In general, NMDs are progressive, impair motor function, and often reduce life expectancy as well as quality of life. Most of the more prevalent NMDs have been first described at the end of the nineteenth century. Although the genetic basis of these disorders has been unraveled during the last century, treatment remained symptomatic or even palliative for many decades. The vast majority of NMDs manifesting in childhood have a genetic basis. Therapeutic agents targeting to treat these conditions can either attempt correcting the genetic defect, or try mitigating the pathophysiological consequences that originate from the genetic error.

NMDs as a whole are not infrequent, but every single one is a rare or orphan disease (prevalence <1 per 1,500 persons in the U.S. and <1 per 2,000 persons in Europe) (1–3). An orphan drug is a pharmaceutical agent developed to treat medical conditions which, because they are so rare, would not be profitable to produce without government assistance (4). Acknowledging the need for better care of patients with rare diseases has led to legislations in the U.S. and in Europe that resulted in tax incentives, enhanced patent protection and marketing rights, and research subsidies, encouraging pharmaceutical companies to develop orphan drugs (4).

These measures together with recent progress in the understanding of pathophysiological mechanisms underlying specific NMDs and in genetic engineering, has resulted in the development of several highly innovative pharmaceutical agents for children with NMDs. This short review gives an overview about recently approved drugs and promising therapeutic agents currently investigated in pre-clinical and clinical trials by focussing on more prevalent pediatric NMDs (Table 1).

**Table 1:** Current status of new therapeutic approaches in pediatric neuromuscular disorders.

**Disease**	**Therapeutic approach**	**Status of development**
**SMA**		
	Gene replacement therapy Onasemnogene abeparvovec	Approved
	Splicing modification Nusinersen Risdiplam	Approved Approved^*^
	Other mode of action SRK-015	Phase 3
**CMT1A**		
	Gene replacement therapy AAV1	Phase 2
	Splicing modification ASOs	Preclinical
	Other mode of action PXT3003 L-Serine	Phase 3 Phase 2
**CMS**		
	Increasing acetylcholine Pyridostigmine 3,4-Diaminonpyridine	Approved Approved
	Modulating channel opening Fluoxetine Quinidine	Approved Approved
	Unknown Salbutamol	Approved
**DM1**		
	Splicing modification Metformin	Phase 2
	Other mode of action Tideglusib Small molecule	Phase 2 Preclinical
**DMD**		
	Gene replacement therapy AAVrh74	Phase 1
	Splicing modification Ataluren Eteplirsen Golodirsen Arbekacin Gentamycin	Approved^*^ Approved^*^ Approved^*^ Phase 2 Phase 1
	Reducing inflammation Prednisone Vamorolone Edasalonexent	Approved Phase 3 Phase 3
	Targeting Cardiomyopathy ACE inhibitors Angiotensin receptor antagonists Beta-blockers	Approved Approved Approved
	Other mode of action Idebenone ACE-031	Phase 3 Phase 2
**LGMD**		
	Gene replacement therapy AAVrh7 beta-sarcoglycan AAVrh74 alpha-sarcoglycan	Phase 2 Phase 2
	AAV1 gamma-sarcoglycan dual AAVrh74 dysferlin	Phase 1 Phase 1
**Congenital myopathies**		
	Gene replacement therapy AAV8 Myotubular myopathy	Phase 2
	Splicing modification DYN10 Dynamin-2 centronuclear myopathy	Phase 2
**Lamin A/C related muscle disease**		
	Splicing modification Exon Skipping	Preclincial
**Pompe**		
	Gene replacement therapy AAV	Preclinical/Phase 1
	Enzyme replacement therapy Lumizyme/Myozyme Neo-GAA ATB200	Approved Phase 3 Phase 4
	Other mode of action Albuterol	Phase 1/2

*SMA, spinal muscular atrophy; CMT1A, Charcot-Marie-tooth 1A; CMS, congenital myasthenic syndromes; DM1, myotonic dystrophy 1; DMD, Duchenne muscular dystrophy; LGMD, limb-girdle muscle dystrophies*.

* *Approved only by the FDA or EMA*.

## Diseases of the Anterior Horn Cell/Spinal Muscular Atrophies (SMAs)

Spinal muscular atrophies are characterized by premature degeneration of the second motor neuron. 5q-associated SMA is by far the most common form with an incidence of about one in 6,000 to 10,000 live births. The phenotype is broad and ranges from infants dying within the first year of life due to respiratory insufficiency to patients showing first symptoms of mild proximal muscle weakness beyond the age of 18 years. The disease is caused by biallelic mutations in the *Survival Motor Neuron* (*SMN1*) gene. *SMN1* encodes the SMN protein that is ubiquitously expressed and essential for proper function of the anterior horn cells in spinal cord and brainstem. About 95% of patients carry homozygous *SMN1* deletions of exon 7 or exons 7 and 8, resulting in a truncated and unstable SMN protein. Humans have 1–8 copies of a paralogous gene, *SMN2*, located next to *SMN1* that differs by five nucleotides. This results in a splicing defect diminishing the SMN protein produced by one copy of *SMN2* to ~10% of the normal value (5, 6). Based on age at onset of clinical symptoms and best motor function 3–5 SMA subtypes (SMA 0, 1, 2, 3 + 4) are distinguished. While biallelic mutations in *SMN1* cause SMA, disease severity is related to the number of *SMN2* copies.

The Food and Drug Administration (FDA) in 2017 and the European Medical Agency (EMA) in 2018 approved nusinersen (Spinraza®), an antisense oligonucleotide that modifies the splicing process of *SMN2*, thereby enhancing the production of stable and functional SMN-protein, for all patients with 5q-associated SMA. Since nusinersen does not pass the blood brain barrier, it has to be administered intrathecally every 4 months following 4 loading doses within the first 2 months. A multicentre placebo-controlled phase 3 study including 121 SMA 1 patients demonstrated that significantly more nusinersen treated subjects were alive and that they had better motor functions at the end of the trial than untreated individuals (7). Similarly, a study with 126 SMA 2 patients aging 2–9 years displayed that subjects receiving nusinersen had significant and clinically meaningful improvement in motor function compared to untreated patients (8). Finally, an observational study with 19 adult SMA 3 patients receiving nusinersen for 10 months showed motor and respiratory improvement (9).

Onasemnogene abeparvovec (Zolgensma®) is a AAV9 vector based gene therapy approved in 2019 by the FDA for children with SMA under the age of 2 years (10), and by the EMA in 2020 for SMA 1 patients and for all SMA subjects with up to 3 *SMN2* copies regardless of their age and weight (11). The Adeno-associated virus subtype 9 (AAV9) is able to cross the blood-brain and to transfect motor neurons. The vector contains a single strain copy of the *SMN1* gene that persists in the cell nucleus as an extrachromosomal episome. The drug is given as an one-time intravenous infusion over 60 minutes. An open-label study including 15 SMA 1 patients showed that all patients were alive and without permanent ventilation by the age of 20 months compared to only 8% of patients from a natural history cohort (12). Moreover, some patients gained motor milestones such as sitting and standing not attained by any patient in the untreated cohort (13). A clinical trial evaluating the safety of intrathecal administration in patients with 3 *SMN2* copies (NCT03381729) is on hold at the time of writing (July, 2020) because of dorsal root ganglia damage observed in non-human primates (14).

Risdiplam (Evrysdi®) is a small molecule also modifying pre-mRNA splicing of *SMN2*. The drug is studied in patients with SMA 1–3 and in pre-symptomatic SMA 1 patients (NCT02908685, NCT02913482, NCT03779334) (15). Risdiplam can be given orally since it penetrates the blood brain barrier (16). Preliminary data from clinical trials in SMA 1 patients and from studies with children and young adults with SMA 2 indicate improved survival and motor function compared to untreated patients (17). Based on these data, Risdiplam has been recently approved by the FDA and is available in Europe in the scope of a compassionate use program for SMA 1 and 2 patients deemed as not suitable for treatment with nusinersen or onasemnogene abeparvovec.

Currently, many SMA 1 patients start treatment with one of the above-mentioned therapeutic agents after they have been diagnosed on clinical grounds. Although the efficacy of these new drugs has been well-documented in clinical trials, improvement of motor function is often modest, and swallowing and respiration remain substantially compromised (7, 18). This stands in sharp contrast to the results of studies with nusinersen and onasemnogene abeparvovec in pre-symptomatic SMA 1 patients, showing that many of them achieve walking, learn to speak, and remain ventilator-free at least within the first years of life (19, 20). These data strongly support inclusion of SMA into new-born screening programs (21–24).

No head-to-head studies comparing the efficacy of nusinersen and onasemnogene abeparvovec are available. An indirect unanchored comparison of the two pivotal trials (7, 12) among symptomatic SMA type 1 infants suggested that onasemnogene abeparvovec may have an efficacy advantage relative to nusinersen (25), but this study has been criticized because of substantial methodological shortcomings (26). The availability of these very expensive new drugs raises many ethical questions such as access to treatment, and start or termination of therapy in patients with advanced disease. Unfortunately, guidelines defining standards of care have been updated for the last time shortly before these new therapies have been approved (27).

Besides these already licensed gene expression modifying drugs, muscle enhancing therapies have been studied. Myostatin is a negative regulator of muscle growth. SRK-015, a monoclonal antibody selectively inhibiting myostatin, has been shown to promote muscle cells growth and differentiation, thereby ameliorating muscle force in SMA mice (28). Safety and tolerability of this approach has been confirmed in a phase I trial (NCT02644777), and a phase II study (NCT03921528) including 58 SMA 2 and 3 children is still ongoing. Preliminary results are expected at the end of this year (29).

## Peripheral Nerve Diseases/Charcot-Marie-Tooth disorders (CMTs)

CMTs are a heterogeneous group of hereditary motor and sensory neuropathies with a prevalence of 1:2,500 (30). Age at onset varies from the neonatal period to adulthood. Clinically, patients have sensory deficits and usually show a slowly progressive distal muscle weakness, and foot and hand deformities. Axonal and demyelinating forms are distinguished, and currently mutations in more than 90 genes are known to be involved (31).

CMT1A is the most frequent form, accounting for about 60% of cases. The disease is caused by a duplication of the *PMP22* gene. Peripheral myelin protein 22 kDa (PMP22) is produced by Schwann cells and has an important role in proliferation and differentiation of myelin. Overexpression of PMP22 protein leads to demyelination and abnormal re-myelination with slowing of nerve conduction velocity, and over time, causes secondary axonal degeneration (32, 33). Different therapeutic approaches are currently under investigation, attempting to regulate PMP22 expression or enhancing myelination (30).

PXT3003 is a combination preparation of three medicaments (3 mg baclofen, 0.35 mg naltrexone, and 105 mg sorbitol) with the aim to reduce the expression of PMP22. A phase 2 placebo-controlled study over 1 year was conducted in 80 patients with CMT1A. Patients in the high-dose arm demonstrated improvement in motor conduction velocities and in the overall neuropathy limitations scale (ONLS) (34). Similarly, a phase 3 study with 323 patients ranging in age from 16 to 65 years taking PXT3003 for 15 months showed an improvement in the 10-m walking test as well as in ONLS in patients receiving the higher dose (NCT02579759) (35). Due to a temporary treatment interruption, the FDA requested an additional pivotal phase 3 trial expected to start in 2021.

Antisense oligonucleotides (ASOs) have been found to effectively suppress PMP22 mRNA in affected nerves in 2 murine CMT1A models. Initiation of ASO treatment restored impressively myelination, motor nerve conduction velocity and compound muscle action potentials almost to levels seen in wild type animals (36). These data demonstrate that strategies to reduce PMP22 have potential as effective therapeutic approaches for CMT1A (37).

Neurotrophin 3 (NT-3) is implicated in the support, survival, and growth of Schwann cells and regeneration of peripheral nerves (38). Preclinical studies with human recombinant NT-3 showed improved axonal regeneration and myelination, which resulted in a subsequent placebo-controlled phase 1/2 study including eight adult patients for 6 months. Patients who received NT-3 subcutaneously experienced a clinical improvement as measured with the Mayo Clinic Neuropathy Score and showed improved regeneration of myelinated fibers in nerve biopsies (39). Because of the limited half-life of subcutaneously administered NT-3 a preclinical AAV mediated NT-3 gene study was conducted in animals, showing improvements in motor function, compound muscle action potentials, and nerve biopsy findings (40). Moreover, a phase 1/2 trial is ongoing with nine patients aging 15 to 35 years treated with intramuscular injections in both legs of AAV1.tMCK.NTF3 (NCT03520751). Results are expected in 2023.

While treatment of CMT1A is still symptomatic and classic pharmacological options have been disappointing (41), a recent randomized trial with L-serine in 18 adults with hereditary sensory autonomic neuropathy (HSAN) showed that the CMT neuropathy score improved significantly in the treated group (42).

## Disorders of the Neuromuscular Junction/Congenital Myasthenic Syndromes (CMS)

CMS include a group of currently 30 genetically distinct entities all sharing the symptoms of fatigable muscle weakness and impaired neurotransmission (43). Application of next generation sequencing techniques has resulted not only in identification of new genes and proteins, but also in a better understanding of the pathophysiology of the neuromuscular junction. This has enabled a more tailored therapeutic approach (44). While drugs like pyridostigmine, an acetylcholine (ACh) esterase inhibitor, often used in tandem with 3,4-diaminonpyridine, a potassium channel blocker increasing ACh release, are more or less effective in some of these conditions, they may worsen symptoms in slow-channel forms, in which prolonged ACh receptor-gated channel opening causes deleterious entry of Ca2+ into the postsynaptic region and consecutive degeneration of post-synaptic structures (43, 44). In addition, treatment with sympathomimetics like salbutamol has been shown to be beneficial in subjects refractory to cholinesterase inhibitors (43). Moreover, ACh receptor open channel blockers, for example, fluoxetine and quinidine, have been shown to be effective in some of the slow channel syndromes (44).

## Primary Diseases of the Skeletal Muscle

### Myotonic Dystrophies

Myotonic dystrophy type 1 and 2 are dominantly inherited, progressive diseases affecting multiple tissues due to unstable repeats in untranslated DNA. Myotonic dystrophy type 1 (DM1) is the most prevalent neuromuscular disorder in adults and has a broad phenotype ranging from onset of myotonia and mild muscle weakness in adulthood to congenital forms characterized by severe muscular hypotonia, generalized muscle weakness, and respiratory failure in neonates due to aggravation of disease severity through successive generations (anticipation) (45). DM1 is caused by an expansion of a CTG repeat sequence in the 3′-UTR of the myotonic dystrophy kinase protein (DMPK) gene. This results in DMPK transcripts with expanded CUG repeats that are retained in the nucleus and form multiple discrete RNA foci, triggering a cascade of toxic effects. Tideglusib is an inhibitor of glycogen synthase kinase 3 beta that has been shown to reduce the amount of toxic CUG-containing RNA in DM1-mice (46). A phase 1/2 study including 16 adolescents and younger adults with DM1 has already been completed, but results are not yet available (NCT02858908), and a double-blind placebo-controlled study assessing safety and efficacy in a smaller group of children with a congenital form is planned (NCT03692312). Metformin is an antidiabetic drug modifying RNA splicing, autophagy, insulin sensitivity, and glycogen synthesis that has been found to have positive effects on mobility and motor function in a small-scale monocentric phase II study (47). The pathophysiology of DM1 is extremely complex, and a bundle of small molecule compounds such as up-regulators of the muscleblind-like (MBNL) splicing factor family, H-RAS pathway inhibitors, transcription inhibitors, and protein kinase modulators has been shown to mitigate DM1 pathogenesis in different experimental systems (48), giving hope that more clinical trials will start in the nearer future (45).

### Duchenne Muscular Dystrophy (DMD)

DMD is caused by mutations in the dystrophin gene, located on Xp21. The dystrophin gene is one of the largest human genes and has 79 exons. The incidence of DMD is 1:3,000 to 1:5,000 male births and the prevalence is 1.3–1.8:10,000 boys. Rarely female carriers with skewed X-inactivation may also develop symptoms (49). Boys typically present with hyperCKemia, motor or global developmental delay, proximal muscle weakness and calf pseudohypertrophy before the age of 5 years. Most patients become wheelchair bound until age 12 years. Scoliosis, dilative cardiomyopathy, and respiratory failure evolve thereafter and result in premature death without assisted ventilation around 20 years of age (50). Recommendations for standards of care have been published (49, 51) and their transformation into clinical practice (e.g., steroid treatment, spinal surgery, non-invasive ventilation) has delayed age at loss of ambulation and substantially increased life expectancy (50, 52, 53).

Dystrophin is part of a protein complex linking the cytoskeleton to the basal lamina, thereby stabilizing the muscle cell membrane. Dystrophin deficiency makes the muscle cell more vulnerable to microtraumas that trigger a cascade of pathophysiological reactions including inflammation, mitochondrial dysfunction, and calcium influx, finally resulting in cell death. Large deletions disrupting the reading frame account for ~65% of mutations, and about 10% of patients carry nonsense mutations resulting in premature termination of the protein synthesis (54). Steroids were the first drugs that have been shown to improve muscle strength and pulmonary function (51, 55). It is supposed that prednisone reduces the inflammatory process as result of the cell membrane breakage, but other effects like enhancing dystrophin expression or lowering calcium influx are also discussed (55). Vamorolone is a synthetic variant of cortisol specifically developed to avoid the side effects of glucocorticoids (56). A phase 3 clinical trial showed that a dosage of 2 mg/kg/die given for 24 weeks was well-tolerated and improved time function tests without negative effects on bone, metabolic, or adrenal function (57). Vamorolone recently received a priority review approval by the FDA. Edasalonexent (CAT-1004) is a small molecule inhibiting the transcription factor NF-κB, which is enhancing muscle degeneration. A phase 1 trial including 17 children has shown good tolerability and reduced expression of NF-kB (NCT02439216) (58). There is an ongoing placebo-controlled phase 3 study to evaluate efficacy and safety of Edasalonexent in 131 pediatric patients (NCT03703882).

There is cumulating evidence that angiotensin converting enzyme inhibitors (ACE inhibitors) reduce the progression rate of dilative cardiomyopathy when initiated early, that is, prior to decline of left ventricular ejection fraction. Similarly, it has been found that the combination of ACE inhibitors or angiotensin receptor antagonists with beta-blockers improves the outcome of patients with established cardiomyopathy (59). Current clinical guidelines recommend an early use of ACE-inhibitors (or angiotensin receptor antagonists for those not tolerating ACE inhibitors) in asymptomatic patients by the age of 10 years (51).

Ataluren (Translarna®) is approved by the EMA, but not yet by the FDA, for treatment of ambulant DMD patients older than age 2 years with nonsense mutations in the dystrophin gene (60). Ataluren is a small molecule acting at the ribosome, that is assumed to read through stop codons and in this way enhances the rate of full-length dystrophin transcripts. The drug is given orally three times a day. A phase 3 study with 115 ambulatory patients treated with ataluren for 48 weeks vs. 115 patients receiving placebo found no significant differences in the 6-min walking test (6MWT), and thus failed the primary endpoint. But a pre-specified analysis in a subgroup of patients with a walking distance of 300–400 m in the 6MWT at baseline demonstrated a significantly lower decline in the walking distance in treated patients (61). Preliminary results of an ongoing prospective study matching the data of 181 DMD patients treated with ataluren for on average 2 years with those of a natural history cohort, suggest a statistically relevant later loss of ambulation in the ataluren group (11 vs. 14.5 years) (62). Further substances assumed to improve read through that have been investigated in phase I and II studies are arbekacin and gentamycin (NCT01918384, NCT00451074).

Exon skipping therapies aim to restore the reading frame in DMD patients with deletions. This allows production of a shortened and defective, but still functional dystrophin protein (63). Among boys with deletions, about 20% patients are amenable to skipping of exon 51, 13% to skipping of exon 53, 12% to skipping of exon 45, and 11% to skipping of exon 44. Eteplirsen (Exondys51®) received an accelerated approval by the FDA in 2016 for the treatment of DMD patients with mutations amenable to skipping of exon 51. The drug is an antisense oligonucleotide that binds to the dystrophin pre-mRNA and supresses correct splicing of exon 51. Results of a phase 2 study showed an increase of dystrophin in muscle biopsies as well as a significantly lower decline in the 6MWT when given to 12 patients for 48 weeks and compared to a placebo group (63). Following up these patients for 3 years and matching their data with those of a natural history cohort also revealed a significant difference in the 6MWT in favor of the eteplirsen group (64). The drug is given intravenously over 30–60 min. Golodirsen (Vyondys53®) has also been approved in December 2019 by the FDA for treatment of DMD patients amenable to skipping of exon 53 (65) after a phase 1/2 clinical trial showed an ~16-fold improvement in dystrophin production (66). The phase 2 and 3 studies are still ongoing (NCT02310906 and NCT02500381). As eteplirsen golodirsen is infused once a week (65). Both drugs are yet not approved by the EMA.

Systemic gene therapy is a promising way to treat DMD. But the size of the dystrophin gene is a major hurdle, since it exceeds the packaging capacity of the AAV vector (67). To overcome this problem different highly abbreviated micro-dystrophins have been invented, and several independent systemic AAV vector mediated gene phase I therapy trials are conducted (NCT03368742, NCT03375164, NCT03362502, NCT03333590). Recently published data on four young DMD patients followed-up for 12 months after a rAAVrh74.MHCK7.micro-dystrophin gene transfer showed that this treatment was well-tolerated, and was associated with robust micro-dystrophin expression, reduced serum CK levels, and functional improvement as measured by the North Star Ambulatory Assessment (68).

Idebenone is an antioxidant that is assumed to improve mitochondrial energy production. In a phase 3 randomized controlled study (DELOS) in DMD patients 10–18 years of age idebenone reduced significantly the loss of respiratory function over a 1-year period, and a *post-hoc* analysis suggested that more patients in the placebo group compared to the idebenone group experienced bronchopulmonary adverse events (69, 70). In addition, the reduced decline of the pulmonary function assessed in a retrospective cohort study (SYROS) from 18 patients was maintained for several years (71). Despite these promising results, the drug has not yet been approved neither by the FDA nor by the EMA.

Preclinical studies exploring myostatin inhibition have shown increased muscle growth as well as reduced fibrosis. ACE-031 is a fusion protein that inhibits myostatin. In a phase 2 clinical trial ACE-031 was injected subcutaneously in 24 patients. This study was stopped because of safety concerns (epistaxis and telangiectasia), but preliminary results showed positive trends concerning distance in the 6MWT and increase of lean body mass (NCT01099761) (72).

Further therapeutic concepts studied in DMD are utrophin upregulation, anti-fibrotic substances, neuronal nitric oxide synthase upregulation and other anti-inflammatory medications (73).

### Limb-Girdle Muscle Dystrophies (LGMDs)

LGMDs are a heterogeneous group of diseases characterized by progressive proximal weakness of the pelvic and/or shoulder muscles. Disease onset is usually after the age of 2 years and the estimated prevalence is 1:100,000 (74). CK concentrations vary from slightly to highly elevated, while muscle biopsy findings range from mildly abnormal to severely dystrophic. Autosomal dominant (LGMD D) and recessive (LGMD R) forms are distinguished. Currently, more than 30 genetically different types are known. Sarcoglycans are proteins that form a tetrameric complex at the muscle cell plasma membrane. This complex stabilizes the association of dystrophin with the dystroglycans and contributes to the stability of the plasma membrane cytoskeleton. Dysferlin or dystrophy-associated fer-1-like protein is encoded by *DYSF*. Several lines of evidence indicate that dysferlin is linked with muscle cell membrane repair. *DYSF* defects can result in different forms of neuromuscular disorders such as Miyoshi myopathy (MM), limb-girdle muscular dystrophy type R2 and Distal myopathy (DM) (75).

#### LGMD R4 (Beta-Sarcoglycanopathy)

Preclinical studies in ß-sarcoglycan deficient mice treated with AAVrh7 containing a β-sarcoglycan transgene targeting to treat skeletal, diaphragm and cardiac muscles demonstrated functional and biochemical improvements (76). Currently, there is an ongoing phase 1/2 clinical trial with six patients ranging in age from 4 to 15 years (NCT03652259). Results from an interim analysis in three patients after 9 months displayed improvement in motor function, reduction of CK, and increase of beta-sarcoglycan expression in the muscle (77). Final results are expected in 2021.

#### LGMD R3 (Alpha-Sarcoglycanopathy)

Preclinical studies in α-sarcoglycan deficient mice treated with systemic AAV containing α-sarcoglycan using a muscle specific promoter showed histological improvement, correction of pseudohypertrophy as well as increase of global activity (78). This prompted a phase 1/2 study that included 3 non-ambulatory patients aging 12 to 14 years, who received an intramuscular injection of AAVrh74 containing an alpha-sarcoglycan transgene. All patients showed a positive gene expression in muscle biopsies performed on week 6, and 3 and 6 months (NCT00494195) (79). Similar results were obtained in two of three patients aging 23 to 43 years old, in whom muscle biopsies were taken 6 months after gene delivery (80). It was supposed that the negative results in the third patient were caused by pre-existing immunity against the used vector (60).

#### LGMD R5 (Gamma-Sarcoglycanopathy)

Preclinical trials in γ-sarcoglycan deficient mice treated with intramuscular AAV containing γ-sarcoglycan with a muscle specific promoter showed histological improvement mainly in those muscles that did not show significant fibrosis (81). In a consecutive phase 1 study 8 out of 9 non-ambulatory patients ranging in age from 12–14 years who received an intramuscular AAV1 injection with a gamma-sarcoglycan transgene showed a positive gene expression in muscle biopsies performed 1 month after application (NCT01344798) (82).

#### LGMD R2 (Dysferlinopathy)

Studies in dysferlin deficient mice treated with systemic dual adeno-associated virus vectors AAVrh74 showed histological and radiological improvement (83). Since *DYSF* is a large gene (55 exons) it has been splintered into two fragments that are packaged into separate AAVrh74 vectors. Both fragments have a 1 kb overlap region that allows the recombination of the two cDNA segments after systemic co-injection (84). Currently, there is an ongoing phase 1 clinical study that includes non-ambulatory adults with LGMDR2 who received intramuscular delivery of AAVrh74 with the aim to explore gene expression after 3 and 6 months (NCT02710500).

### Congenital Myopathies

#### Myotubular Myopathy

X-linked Myotubular Myopathy (XLMTM) is a rare congenital myopathy characterized by severe muscle weakness, respiratory failure and early death. Mortality rate is estimated to be 50% in the first 18 months of life. The disease is caused by mutations in the *MTM1* gene that lead to absence or dysfunction of myotubularin, a protein that is necessary for normal development, maturation, and function of skeletal muscle cells. The disease affects ~1 in 50,000 new-born males. In a study with XLMTM dogs, intravenous administration of a recombinant AAV8 vector expressing canine myotubularin at 10 weeks of age demonstrated impressively that this treatment was well-tolerated, prolonged lifespan, and corrected the skeletal muscle phenotype in a dose-dependent manner. This prompted a phase 1/2 study (NCT03199469) in children with XLMTM ranging in age from 0–5 years. While the first six patients dosed at 1 × ·10^14^ genome copies/kg also showed very encouraging results, all 3 patients administered a 3-fold higher dose (3 × 10^14^) experienced severe hepatotoxicity and two of them died (85). Further development of this product is currently on hold pending further evaluation of these serious adverse events (86).

#### Dynamin-2 Related Centronuclear Myopathy

Mutations in *DNM2* encoding dynamin-2 cause autosomal dominant centronuclear myopathy, which is associated with variable muscle weakness and wasting (87). In *DNM2*-mutated mice, weekly intrapleural injections of ASOs targeting DNM2 for 5 weeks corrected muscle mass, histopathology, and muscle ultrastructure (88). These findings prompted an ongoing phase 1/2 study with DYN101, a synthetically manufactured constrained ethyl gapmer ASO directed against DNM2 pre-mRNA in 18 adolescents and young adults (NCT04033159).

### Lamin A/C Related Muscle Disease

LMNA-related disorders are caused by mutations in the LMNA gene, encoding the nuclear envelope proteins lamin A and C by alternative splicing. LMNA mutations are linked with a wide range of disease phenotypes such as neuromuscular, cardiac and metabolic disorders to premature aging syndromes. Neuromuscular phenotypes include LMNA-related muscular dystrophy, autosomal dominant Emery-Dreifuss muscular dystrophy, and congenital muscular dystrophy. Apart from symptomatic treatment including the use of steroids amelioration of pathogenesis by exon skipping has been proposed as a potential treatment strategy (89).

### Pompe Disease

Pompe disease (glycogen storage disease type 2) is caused by biallelic mutations of the acid alpha-glucosidase (GAA) gene. This results in deficiency of the lysosomal enzyme GAA and impaired autophagy. Severity of disease depends on the amount of residual enzyme activity. Two types are distinguished, infantile and late onset Pompe disease (IOPD/LOPD). The incidence of IOPD is about 1:140,000 and that of all types amounts to ~1:40,000 in Europe. In classic IOPD, GAA activity is <1%. Contrary to milder forms with later onset that are characterized mainly by a progressive proximal myopathy with early respiratory involvement, this causes marked accumulation of glycogen not only in skeletal muscle, but also in heart and other tissues. Affected patients present with CK elevation, hypertrophic cardiomyopathy, failure to thrive, muscular hypotonia and axial muscle weakness during the first 6 months of life. IOPD is rapidly progressive and the majority of untreated subjects die within the first year of life due to a combination of ventilatory and cardiac failure without achieving any motor mile stone such as turning, sitting, or standing. Survival beyond the age of 18 months is exceptional. Although GAA activity is <1% in all IOPD patients, two groups have to be differentiated. Patients may synthesize a non-functional form of GAA or are completely unable to form any kind of native enzyme. The former patients are designated as cross-reactive immunological material (CRIM)-positive, whereas the latter are classified as CRIM-negative (90–92).

An enzyme replacement therapy (ERT) with recombinant human alpha-glucosidase (Lumizyme®/Myozyme®) was approved in 2006 by the FDA and the EMA. The recombinant enzyme has to be administered intravenously every 2 weeks over about 4 h. In the pivotal phase 3 study including 18 infants diagnosed before 6 months of age, all patients were alive and seven walked independently after 12 months of ERT (93). In a follow-up extension study over 3 years 13 patients were still alive and 6 remained able to walk (94). In addition, a placebo-controlled phase 3 study was performed in 90 ambulant LOPD patients about 18 months. This showed that treated patients had significantly better motor function (measured by the 6MWT) as well as stabilized pulmonary function (95).

The positive results of the pivotal IOPD trials have been confirmed by several real world studies, but it has been recognized over the years that the response to ERT is imperfect and that patients respond differently. A beginning of ERT as early as possible and immunomodulation in CRIM-negative subjects to avoid antibody formation against the recombinant enzyme have been identified as important factors to improve outcome, but morbidity and mortality are still high. Moreover, prolonged survival of IOPD patients has resulted in a new phenotype with variable residual muscle weakness and worsening of motor function after some years of ERT, hearing impairment, oropharyngeal and facial weakness causing speech and swallowing difficulties as well as neurocognitive, respiratory and orthopedic problems (96, 97). Strategies to further improve outcome in IOPD and also LOPD focus on manufacturing improved enzyme versions that allow a better uptake into the muscle cell, and developing gene therapies. Neo-GAA (avalglucosidasa alfa), a modified recombinant human GAA with higher affinity to the mannose-6-phosphate (M6P) receptor, is currently tested in a phase 3 study and final results are expected soon (NCT02782741). ATB200 is another rhGAA with a higher content of M6P and bis-M6P glycan residues that is tested in a clinical trial in association with a pharmacological chaperone. Albuterol has also been investigated as an add-on therapy which may enhance the lysosomal uptake of hrGAA (98). Moreover, there are several pre-clinical and early clinical *ex-vivo* and *in-vivo* gene therapy trials targeting different tissues with variable transgenes (for review see Ronzitti et al. ATM 2019) (99). To date, it is not clear which approach will finally be best suited for this complex disease.

## Conclusions

Several new therapeutic options have become available for the treatment of pediatric NMDs in the last years, and multiple others are currently studied in pre-clinical and clinical trials. While some diseases have now become principally treatable, many others are still waiting for the major breakthrough. The new therapeutic options in SMA 1 and IOPD are examples for drugs that have transformed rapidly progressive lethal diseases into more chronic conditions. But it has to be kept in mind that patients treated this way are not cured. Moreover, both conditions show that success and efficacy of these new therapies depend on the time point of their application and the clinical status of the patients. Pompe disease was the first NMD that became principally treatable. One lesson to be learned from the use of ERT in IOPD since almost 15 years is that problems emerged totally unexpected before. It does not take a crystal ball to see that similar things will happen in other diseases. Nevertheless, the development of new, highly innovative drugs has heralded a new era in the treatment of pediatric NMDs.

## Author Contributions

MF-B conceived and designed the work and draft the article. AH designed the work and performed a critical revision of the article. All authors contributed to the final manuscript.

## Conflict of Interest

MF-B received honoraria for participation in advisory boards and consultancy fees from Biogen, Roche, and PTC. The remaining author declares that the research was conducted in the absence of any commercial or financial relationships that could be construed as a potential conflict of interest.

## References

[1] European Union Regulation (EC) N°141/2000 of the European Parliament and of the Council of 16 December 1999 on Orphan Medicinal Products. (2000). Available online at: http://data.europa.eu/eli/reg/2000/141/oj (access October 10, 2020).

[2] Baldovino S Moliner AM Taruscio D Daina E Roccatello D. Rare diseases in Europe: from a wide to a local perspective. Isr Med Assoc J. (2016) 18:359–63. 27468531

[3] Deenen JCW Horlings CGC Verschuuren JJGM Verbeek ALM van Engelen BGM. The epidemiology of neuromuscular disorders: a comprehensive overview of the literature. J Neuromuscul Dis. (2015) 2:73–85. 10.3233/JND-140045 28198707

[4] Giannuzzi V Conte R Landi A Ottomano SA Bonifazi D Baiardi P. Orphan medicinal products in Europe and United States to cover needs of patients with rare diseases: an increased common effort is to be foreseen. Orphanet J Rare Dis. (2017) 12:64. 10.1186/s13023-017-0617-1 28372595 PMC5376695

[5] Lunn MR Wang CH. Spinal muscular atrophy. Lancet. (2008) 371:2120–33. 10.1016/S0140-6736(08)60921-6 18572081

[6] Pechmann A Kirschner J. Diagnosis and new treatment avenues in spinal muscular atrophy. Neuropediatrics. (2017) 48:273–81. 10.1055/s-0037-1603517 28571100

[7] Finkel RS Mercuri E Darras BT Connolly AM Kuntz NL Kirschner J. Nusinersen versus sham control in infantile-onset spinal muscular atrophy. N Engl J Med. (2017) 377:1723–32. 10.1056/NEJMoa1702752 29091570

[8] Mercuri E Darras BT Chiriboga CA Day JW Campbell C Connolly AM. Nusinersen versus sham control in later-onset spinal muscular atrophy. N Engl J Med. (2018) 378:625–35. 10.1056/NEJMoa1710504 29443664

[9] Walter MC Wenninger S Thiele S Stauber J Hiebeler M Greckl E. Safety and treatment effects of nusinersen in longstanding adult 5q-SMA type 3—a prospective observational study. J Neuromuscul Dis. (2019) 6:453–65. 10.3233/JND-190416 31594243 PMC6918909

[10] Hoy SM. Onasemnogene abeparvovec: first global approval. Drugs. (2019) 79:1255–62. 10.1007/s40265-019-01162-5 31270752

[11] Buckingham L New Gene Therapy to Treat Spinal Muscular Atrophy (corrected) (2020). *European Medicines Agency* [zitiert 2. Mai 2020]. Available online at: https://www.ema.europa.eu/en/news/new-gene-therapy-treat-spinal-muscular-atrophy-corrected (access October 10, 2020).

[12] Mendell JR Al-Zaidy S Shell R Arnold WD Rodino-Klapac LR Prior TW. Single-dose gene-replacement therapy for spinal muscular atrophy. N Engl J Med. (2017) 377:1713–22. 10.1056/NEJMoa1706198 29091557

[13] Al-Zaidy SA Kolb SJ Lowes L Alfano LN Shell R Church KR. AVXS-101 (Onasemnogene Abeparvovec) for SMA1: comparative study with a prospective natural history cohort. J Neuromuscul Dis. (2019) 6:307–17. 10.3233/JND-190403 31381526

[14] Kirschner J Butoianu N Goemans N Haberlova J Kostera-Pruszczyk A Mercuri E. European ad-hoc consensus statement on gene replacement therapy for spinal muscular atrophy. Eur J Paediatr Neurol. (2020) 28:38–43. 10.1016/j.ejpn.2020.07.001 32763124 PMC7347351

[15] Sturm S Günther A Jaber B Jordan P Al Kotbi N Parkar N. A phase 1 healthy male volunteer single escalating dose study of the pharmacokinetics and pharmacodynamics of risdiplam (RG7916, RO7034067), a SMN2 splicing modifier. Br J Clin Pharmacol. (2019) 85:181–93. 10.1111/bcp.13786 30302786 PMC6303280

[16] Poirier A Weetall M Heinig K Bucheli F Schoenlein K Alsenz J. Risdiplam distributes and increases SMN protein in both the central nervous system and peripheral organs. Pharmacol Res Perspect. (2018) 6:e00447. 10.1002/prp2.447 30519476 PMC6262736

[17] Baranello G Servais L Day J Deconinck N Mercuri E Klein A P.353FIREFISH Part 1: 16-month safety and exploratory outcomes of risdiplam (RG7916) treatment in infants with type 1 spinal muscular atrophy. Neuromuscul Disord. (2019) 29:S184 10.1016/j.nmd.2019.06.515

[18] Day J Chiriboga C Darras B Finkel R Connolly A Iannacconne S Onasemnogene Abeparvovec-xioi gene-replacement therapy for spinal muscular atrophy type 1 (SMA1): phase 3 US study (STR1VE) update (1828). Neurology. (2020) 94(Suppl. 15).

[19] De Vivo DC Bertini E Swoboda KJ Hwu W-L Crawford TO Finkel RS. Nusinersen initiated in infants during the presymptomatic stage of spinal muscular atrophy: interim efficacy and safety results from the phase 2 nurture study. Neuromuscul Disord. (2019) 29:842–56. 10.1016/j.nmd.2019.09.007 31704158 PMC7127286

[20] Lowes LP Alfano LN Arnold WD Shell R Prior TW McColly M. Impact of age and motor function in a phase 1/2a study of infants with SMA type 1 receiving single-dose gene replacement therapy. Pediatr Neurol. (2019) 98:39–45. 10.1016/j.pediatrneurol.2019.05.005 31277975

[21] Boemer F Caberg J-H Dideberg V Dardenne D Bours V Hiligsmann M. Newborn screening for SMA in Southern Belgium. Neuromuscul Disord. (2019) 29:343–9. 10.1016/j.nmd.2019.02.003 31030938

[22] Glascock J Sampson J Haidet-Phillips A Connolly A Darras B Day J. Treatment algorithm for infants diagnosed with spinal muscular atrophy through newborn screening. J Neuromuscul Dis. (2018) 5:145–58. 10.3233/JND-180304 29614695 PMC6004919

[23] Vill K Kölbel H Schwartz O Blaschek A Olgemöller B Harms E. One year of newborn screening for SMA—results of a German pilot project. J Neuromuscul Dis. (2019) 6:503–15. 10.3233/JND-190428 31594245 PMC6918901

[24] Govoni A Gagliardi D Comi GP Corti S. Time is motor neuron: therapeutic window and its correlation with pathogenetic mechanisms in spinal muscular atrophy. Mol Neurobiol. (2018) 55:6307–18. 10.1007/s12035-017-0831-9 29294245

[25] Dabbous O Maru B Jansen JP Lorenzi M Cloutier M Guérin A. Survival, motor function, and motor milestones: comparison of AVXS-101 relative to nusinersen for the treatment of infants with spinal muscular atrophy type 1. Adv Ther. (2019) 36:1164–76. 10.1007/s12325-019-00923-8 30879249 PMC6824368

[26] Sandrock AW Farwell W. Comparisons between separately conducted clinical trials: letter to the editor regarding Dabbous O, Maru B, Jansen JP, Lorenzi M, Cloutier M, Guérin A, et al. Adv Ther. (2019) 36:1164–76. 10.1007/s12325-019-01087-1 31512142 PMC6822795

[27] Finkel RS Mercuri E Meyer OH Simonds AK Schroth MK Graham RJ. Diagnosis and management of spinal muscular atrophy: part 2: pulmonary and acute care; medications, supplements and immunizations; other organ systems; and ethics. Neuromuscul Disord. (2018) 28:197–207. 10.1016/j.nmd.2017.11.004 29305137

[28] Long KK O'Shea KM Khairallah RJ Howell K Paushkin S Chen KS. Specific inhibition of myostatin activation is beneficial in mouse models of SMA therapy. Hum Mol Genet. (2019) 28:1076–89. 10.1093/hmg/ddy382 30481286 PMC6423420

[29] Messina S Sframeli M New treatments in spinal muscular atrophy: positive results and new challenges. J Clin Med. (2020) 9:2222 10.3390/jcm9072222 PMC7408870 32668756

[30] Morena J Gupta A Hoyle JC. Charcot-Marie-tooth: from molecules to therapy. Int J Mol Sci. (2019) 20:3419. 10.3390/ijms20143419 31336816 PMC6679156

[31] Mathis S Goizet C Tazir M Magdelaine C Lia A-S Magy L. Charcot–Marie–tooth diseases: an update and some new proposals for the classification. J Med Genet. (2015) 52:681. 10.1136/jmedgenet-2015-103272 26246519

[32] van Paassen BW van der Kooi AJ van Spaendonck-Zwarts KY Verhamme C Baas F de Visser M. PMP22 related neuropathies: Charcot-Marie-tooth disease type 1A and hereditary neuropathy with liability to Pressure Palsies. Orphanet J Rare Dis. (2014) 9:38. 10.1186/1750-1172-9-38 24646194 PMC3994927

[33] Watila MM Balarabe SA. Molecular and clinical features of inherited neuropathies due to PMP22 duplication. J Neurol Sci. (2015) 355:18–24. 10.1016/j.jns.2015.05.037 26076881

[34] Attarian S Vallat J-M Magy L Funalot B Gonnaud P-M Lacour A. An exploratory randomised double-blind and placebo-controlled phase 2 study of a combination of baclofen, naltrexone and sorbitol (PXT3003) in patients with Charcot-Marie-Tooth disease type 1A. Orphanet J Rare Dis. (2014) 9:199. 10.1186/s13023-014-0199-0 25519680 PMC4311411

[35] Thomas F Boutalbi Y Fitoussi S Rinaudo P Bertrand V Hajj R Efficacy ANS safety of PXT3003 in patients with Charcot-Marie-tooth disease type1A: results of Pleo-Charcot–Marie–tooth: an international pivotal phase III trial. Muscle Nerve. (2019) 60(S1):S46.

[36] Zhao HT Damle S Ikeda-Lee K Kuntz S Li J Mohan A. PMP22 antisense oligonucleotides reverse Charcot-Marie-tooth disease type 1A features in rodent models. J Clin Invest. (2018) 128:359–68. 10.1172/JCI96499 29202483 PMC5749515

[37] Shy ME. Antisense oligonucleotides offer hope to patients with Charcot-Marie-tooth disease type 1A. J Clin Invest. (2018) 128:110–2. 10.1172/JCI98617 29199996 PMC5749496

[38] Sahenk Z. Neurotrophins and peripheral neuropathies. Brain Pathol. (2006) 16:311–9. 10.1111/j.1750-3639.2006.00038.x 17107601 PMC8096058

[39] Sahenk Z Nagaraja HN McCracken BS King WM Freimer ML Cedarbaum JM. NT-3 promotes nerve regeneration and sensory improvement in CMT1A mouse models and in patients. Neurology. (2005) 65:681–9. 10.1212/01.WNL.0000171978.70849.c5 16157899

[40] Sahenk Z Galloway G Clark KR Malik V Rodino-Klapac LR Kaspar BK. AAV1.NT-3 gene therapy for Charcot–Marie–tooth neuropathy. Mol Ther. (2014) 22:511–21. 10.1038/mt.2013.250 24162799 PMC3944324

[41] Boutary S Echaniz-Laguna A Adams D Loisel-Duwattez J Schumacher M Massaad C. Treating PMP22 gene duplication-related Charcot-Marie-Tooth disease: the past, the present and the future. Transl Res. (2020) S1931–5244:30175–4. 10.1016/j.trsl.2020.07.006 32693030

[42] Fridman V Suriyanarayanan S Novak P David W Macklin EA McKenna-Yasek D. Randomized trial of l-serine in patients with hereditary sensory and autonomic neuropathy type 1. Neurology. (2019) 92:e359–70. 10.1212/WNL.0000000000006811 30626650 PMC6345118

[43] Nicole S Azuma Y Bauché S Eymard B Lochmüller H Slater C. Congenital myasthenic syndromes or inherited disorders of neuromuscular transmission: recent discoveries and open questions. J Neuromuscul Dis. (2017) 4:269–84. 10.3233/JND-170257 29125502 PMC5701762

[44] Vanhaesebrouck AE Beeson D. The congenital myasthenic syndromes: expanding genetic and phenotypic spectrums and refining treatment strategies. Curr Opin Neurol. (2019) 32:696–703. 10.1097/WCO.0000000000000736 31361628 PMC6735524

[45] Turner C Hilton-Jones D. Myotonic dystrophy: diagnosis, management and new therapies. Curr Opin Neurol. (2014) 27:599–606. 10.1097/WCO.0000000000000128 25121518

[46] Timchenko L. Correction of RNA-binding protein CUGBP1 and GSK3β signaling as therapeutic approach for congenital and adult myotonic dystrophy type 1. Int J Mol Sci. (2019) 21:94. 10.3390/ijms21010094 31877772 PMC6982105

[47] Bassez G Audureau E Hogrel J-Y Arrouasse R Baghdoyan S Bhugaloo H. Improved mobility with metformin in patients with myotonic dystrophy type 1: a randomized controlled trial. Brain. (2018) 141:2855–65. 10.1093/brain/awy231 30169600

[48] López-Morató M Brook JD Wojciechowska M. Small molecules which improve pathogenesis of myotonic dystrophy type 1. Front Neurol. (2018) 9:349. 10.3389/fneur.2018.00349 29867749 PMC5968088

[49] Bushby K Finkel R Birnkrant DJ Case LE Clemens PR Cripe L. Diagnosis and management of Duchenne muscular dystrophy, part 1: diagnosis, and pharmacological and psychosocial management. Lancet Neurol. (2010) 9:77–93. 10.1016/S1474-4422(09)70271-6 19945913

[50] McDonald CM Henricson EK Abresch RT Han JJ Escolar DM Florence JM. The cooperative international neuromuscular research group Duchenne natural history study—a longitudinal investigation in the era of glucocorticoid therapy: design of protocol and the methods used. Muscle Nerve. (2013) 48:32–54. 10.1002/mus.23807 23677550 PMC4147958

[51] Birnkrant DJ Bushby K Bann CM Apkon SD Blackwell A Brumbaugh D. Diagnosis and management of Duchenne muscular dystrophy, part 1: diagnosis, and neuromuscular, rehabilitation, endocrine, and gastrointestinal and nutritional management. Lancet Neurol. (2018) 17:251–67. 10.1016/S1474-4422(18)30024-3 29395989 PMC5869704

[52] Landfeldt E Thompson R Sejersen T McMillan HJ Kirschner J Lochmüller H. Life expectancy at birth in Duchenne muscular dystrophy: a systematic review and meta-analysis. Eur J Epidemiol. (2020) 35:643–53. 10.1007/s10654-020-00613-8 32107739 PMC7387367

[53] McDonald CM Henricson EK Abresch RT Duong T Joyce NC Hu F. Long-term effects of glucocorticoids on function, quality of life, and survival in patients with Duchenne muscular dystrophy: a prospective cohort study. Lancet. (2018) 391:451–61. 10.1016/S0140-6736(17)32160-8 29174484

[54] Aartsma-Rus A Ginjaar IB Bushby K. The importance of genetic diagnosis for Duchenne muscular dystrophy. J Med Genet. (2016) 53:145–51. 10.1136/jmedgenet-2015-103387 26754139 PMC4789806

[55] Moxley RT Ashwal S Pandya S Connolly A Florence J Mathews K. Practice parameter: corticosteroid treatment of Duchenne dystrophy: report of the Quality Standards Subcommittee of the American Academy of Neurology and the Practice Committee of the Child Neurology Society. Neurology. (2005) 64:13–20. 10.1212/01.WNL.0000148485.00049.B7 15642897

[56] Hoffman EP Riddle V Siegler MA Dickerson D Backonja M Kramer WG et sl. Phase 1 trial of vamorolone, a first-in-class steroid, shows improvements in side effects via biomarkers bridged to clinical outcomes. Steroids. (2018) 134:43–52. 10.1016/j.steroids.2018.02.010 29524454 PMC6136660

[57] Hoffman EP Schwartz BD Mengle-Gaw LJ Smith EC Castro D Mah JK. Vamorolone trial in Duchenne muscular dystrophy shows dose-related improvement of muscle function. Neurology. (2019) 93:e1312–23. 10.1212/WNL.0000000000008168 31451516 PMC7011869

[58] Finanger E Vandenborne K Finkel RS Lee Sweeney H Tennekoon G Yum S. Phase 1 study of edasalonexent (CAT-1004), an oral NF-κB inhibitor, in pediatric patients with Duchenne muscular dystrophy. J Neuromuscul Dis. (2019) 6:43–54. 10.3233/JND-180341 30452422 PMC6398836

[59] El-Aloul B Altamirano-Diaz L Zapata-Aldana E Rodrigues R Malvankar-Mehta MS Nguyen C-T. Pharmacological therapy for the prevention and management of cardiomyopathy in Duchenne muscular dystrophy: a systematic review. Neuromuscul Disord. (2017) 27:4–14. 10.1016/j.nmd.2016.09.019 27815032

[60] Ryan NJ. Ataluren: first global approval. Drugs. (2014) 74:1709–14. 10.1007/s40265-014-0287-4 25193627

[61] McDonald CM Campbell C Torricelli RE Finkel RS Flanigan KM Goemans N. Ataluren in patients with nonsense mutation Duchenne muscular dystrophy (ACT DMD): a multicentre, randomised, double-blind, placebo-controlled, phase 3 trial. Lancet. (2017) 390:1489–98. 10.1016/S0140-6736(17)31611-2 28728956

[62] Mercuri E Muntoni F Osorio AN Tulinius M Buccella F Morgenroth LP. Safety and effectiveness of ataluren: comparison of results from the STRIDE Registry and CINRG DMD natural history study. J Comp Eff Res. (2020) 9:341–60. 10.2217/cer-2019-0171 31997646 PMC7610147

[63] Mendell JR Rodino-Klapac LR Sahenk Z Roush K Bird L Lowes LP. Eteplirsen for the treatment of Duchenne muscular dystrophy. Ann Neurol. (2013) 74:637–47. 10.1002/ana.23982 23907995

[64] Mendell JR Goemans N Lowes LP Alfano LN Berry K Shao J. Longitudinal effect of eteplirsen versus historical control on ambulation in Duchenne muscular dystrophy. Ann Neurol. (2016) 79:257–71. 10.1002/ana.24555 26573217 PMC5064753

[65] Heo Y-A. Golodirsen: first approval. Drugs. (2020) 80:329–33. 10.1007/s40265-020-01267-2 32026421

[66] Frank DE Schnell FJ Akana C El-Husayni SH Desjardins CA Morgan J. Increased dystrophin production with golodirsen in patients with Duchenne muscular dystrophy. Neurology. (2020) 94:e2270–82. 10.1212/WNL.0000000000009233 32139505 PMC7357297

[67] Duan D. Systemic AAV micro-dystrophin gene therapy for Duchenne muscular dystrophy. Mol Ther. (2018) 26:2337–56. 10.1016/j.ymthe.2018.07.011 30093306 PMC6171037

[68] Mendell JR Sahenk Z Lehman K Nease C Lowes LP Miller NF. Assessment of systemic delivery of rAAVrh74.MHCK7.micro-dystrophin in children with Duchenne muscular dystrophy: a nonrandomized controlled trial. JAMA Neurol. (2020) 77:1–10. 10.1001/jamaneurol.2020.1484 32539076 PMC7296461

[69] Buyse GM Goemans N van den Hauwe M Thijs D de Groot IJM Schara U. Idebenone as a novel, therapeutic approach for Duchenne muscular dystrophy: results from a 12 month, double-blind, randomized placebo-controlled trial. Neuromuscul Disord. (2011) 21:396–405. 10.1016/j.nmd.2011.02.016 21435876

[70] McDonald CM Meier T Voit T Schara U Straathof CSM D'Angelo MG. Idebenone reduces respiratory complications in patients with Duchenne muscular dystrophy. Neuromuscul Disord. (2016) 26:473–80. 10.1016/j.nmd.2016.06.257 27238057

[71] Servais L Straathof CSM Schara U Klein A Leinonen M Hasham S. Long-term data with idebenone on respiratory function outcomes in patients with Duchenne muscular dystrophy. Neuromuscul Disord. (2020) 30:5–16. 10.1016/j.nmd.2019.10.008 31813614

[72] Campbell C McMillan HJ Mah JK Tarnopolsky M Selby K McClure T. Myostatin inhibitor ACE-031 treatment of ambulatory boys with Duchenne muscular dystrophy: results of a randomized, placebo-controlled clinical trial. Muscle Nerve. (2017) 55:458–64. 10.1002/mus.25268 27462804

[73] Dowling JJ D Gonorazky H Cohn RD Campbell C. Treating pediatric neuromuscular disorders: the future is now. Am J Med Genet A. (2018) 176:804–41. 10.1002/ajmg.a.38418 28889642 PMC5900978

[74] Liewluck T Milone M. Untangling the complexity of limb-girdle muscular dystrophies. Muscle Nerve. (2018) 58:167–77. 10.1002/mus.26077 29350766

[75] Fanin M Angelini C. Progress and challenges in diagnosis of dysferlinopathy. Muscle Nerve. (2016) 54:821–35. 10.1002/mus.25367 27501525

[76] Pozsgai ER Griffin DA Heller KN Mendell JR Rodino-Klapac LR. Systemic AAV-mediated β-sarcoglycan delivery targeting cardiac and skeletal muscle ameliorates histological and functional deficits in LGMD2E mice. Mol Ther. (2017) 25:855–69. 10.1016/j.ymthe.2017.02.013 28284983 PMC5383645

[77] Estepan I Sarepta Therapeutics Announces Positive Functional Results from the SRP-9003 (MYO-101) Gene Therapy Trial to Treat Limb-Girdle Muscular Dystrophy Type 2E, or Beta-Sarcoglycanopathy | Sarepta Therapeutics, Inc. Investorrelations. (2019). Available online at: https://investorrelations.sarepta.com/news-releases/news-release-details/sarepta-therapeutics-announces-positive-functional-results-srp (accessed June 13, 2020).

[78] Fougerousse F Bartoli M Poupiot J Arandel L Durand M Guerchet N Phenotypic correction of α-sarcoglycan deficiency by intra-arterial injection of a muscle-specific serotype 1 rAAV vector. Mol Ther. (2007) 15:53–61. 10.1038/sj.mt.6300022 28182933

[79] Mendell JR Rodino-Klapac LR Rosales-Quintero X Kota J Coley BD Galloway G. Limb-girdle muscular dystrophy type 2D gene therapy restores alpha-sarcoglycan and associated proteins. Ann Neurol. (2009) 66:290–7. 10.1002/ana.21732 19798725 PMC6014624

[80] Mendell JR Rodino-Klapac LR Rosales XQ Coley BD Galloway G Lewis S. Sustained alpha-sarcoglycan gene expression after gene transfer in limb-girdle muscular dystrophy, type 2D. Ann Neurol. (2010) 68:629–38. 10.1002/ana.22251 21031578 PMC2970162

[81] Cordier L Hack AA Scott MO Barton-Davis ER Gao G Wilson JM. Rescue of skeletal muscles of gamma-sarcoglycan-deficient mice with adeno-associated virus-mediated gene transfer. Mol Ther. (2000) 1:119–29. 10.1006/mthe.1999.0019 10933922

[82] Herson S Hentati F Rigolet A Behin A Romero NB Leturcq F. A phase I trial of adeno-associated virus serotype 1-γ-sarcoglycan gene therapy for limb girdle muscular dystrophy type 2C. Brain. (2012) 135(Pt 2):483–92. 10.1093/brain/awr342 22240777

[83] Potter RA Griffin DA Sondergaard PC Johnson RW Pozsgai ER Heller KN. Systemic delivery of dysferlin overlap vectors provides long-term gene expression and functional improvement for dysferlinopathy. Hum Gene Ther. (2018) 29:749–62. 10.1089/hum.2017.062 28707952 PMC6066196

[84] Sondergaard PC Griffin DA Pozsgai ER Johnson RW Grose WE Heller KN. AAV.Dysferlin overlap vectors restore function in dysferlinopathy animal models. Ann Clin Transl Neurol. (2015) 2:256–70. 10.1002/acn3.172 25815352 PMC4369275

[85] Annoussamy M Lilien C Gidaro T Gargaun E Chê V Schara U. X-linked myotubular myopathy: a prospective international natural history study. Neurology. (2019) 92:e1852–67. 10.1212/WNL.0000000000007319 30902907 PMC6550499

[86] Wilson JM Flotte TR. Moving forward after two deaths in a gene therapy trial of myotubular myopathy. Hum Gene Ther. (2020) 31:695–6. 10.1089/hum.2020.182 32605399

[87] Zhao M Maani N Dowling JJ. Dynamin 2 (DNM2) as cause of, and modifier for, human neuromuscular disease. Neurotherapeutics. (2018) 15:966–75. 10.1007/s13311-018-00686-0 30426359 PMC6277281

[88] Buono S Ross JA Tasfaout H Levy Y Kretz C Tayefeh L. Reducing dynamin 2 (DNM2) rescues DNM2-related dominant centronuclear myopathy. Proc Natl Acad Sci USA. (2018) 115:11066–71. 10.1073/pnas.1808170115 30291191 PMC6205463

[89] Scharner J Figeac N Ellis JA Zammit PS. Ameliorating pathogenesis by removing an exon containing a missense mutation: a potential exon-skipping therapy for laminopathies. Gene Ther. (2015) 22:503–15. 10.1038/gt.2015.8 25832542

[90] American Association of Neuromuscular & Electrodiagnostic Medicine. Diagnostic criteria for late-onset (childhood and adult) Pompe disease. Muscle Nerve. (2009) 40:149–60. 10.1002/mus.21393 19533647

[91] Cupler EJ Berger KI Leshner RT Wolfe GI Han JJ Barohn RJ. Consensus treatment recommendations for late-onset Pompe disease. Muscle Nerve. (2012) 45:319–33. 10.1002/mus.22329 22173792 PMC3534745

[92] van der Ploeg AT Reuser AJJ. Pompe's disease. Lancet. (2008) 372:1342–53. 10.1016/S0140-6736(08)61555-X 18929906

[93] Kishnani PS Corzo D Nicolino M Byrne B Mandel H Hwu WL. Recombinant human acid [alpha]-glucosidase: major clinical benefits in infantile-onset Pompe disease. Neurology. (2007) 68:99–109. 10.1212/01.wnl.0000251268.41188.04 17151339

[94] Kishnani PS Corzo D Leslie ND Gruskin D Van der Ploeg A Clancy JP. Early treatment with alglucosidase alpha prolongs long-term survival of infants with Pompe disease. Pediatr Res. (2009) 66:329–35. 10.1203/PDR.0b013e3181b24e94 19542901 PMC3129995

[95] van der Ploeg AT Clemens PR Corzo D Escolar DM Florence J Groeneveld GJ A randomized study of alglucosidase alfa in late-onset Pompe's disease. N Engl J Med. (2010) 362:1396–406. 10.1056/NEJMoa0909859 20393176

[96] Kohler L Puertollano R Raben N Pompe disease: from basic science to therapy. Neurotherapeutics. (2018) 15:928–42. 10.1007/s13311-018-0655-y 30117059 PMC6277280

[97] Hahn A Schänzer A Long-term outcome and unmet needs in infantile-onset Pompe disease. Ann Transl Med. (2019) 7:283 10.21037/atm.2019.04.70 31392195 PMC6642934

[98] Koeberl DD Case LE Desai A Smith EC Walters C Han S-O Improved muscle function in a phase I/II clinical trial of albuterol in Pompe disease. Mol Genet Metab. (2020) 129:67–72. 10.1016/j.ymgme.2019.12.008 31839530

[99] Ronzitti G Collaud F Laforet P Mingozzi F Progress and challenges of gene therapy for Pompe disease. Ann Transl Med. (2019) 7:287 10.21037/atm.2019.04.67 31392199 PMC6642941

